# Seeing without the Occipito-Parietal Cortex: Simultagnosia as a Shrinkage of the Attentional Visual Field

**DOI:** 10.1155/2004/836830

**Published:** 2004-06-09

**Authors:** François Michel, Marie-Anne Henaff

**Affiliations:** ^1^Institut des Sciences CognitivesCNRS. LyonFrance; ^2^U 280 INSERMLyonFrance

**Keywords:** Balint’s syndrome, visual attention, attentional visual field, bilateral neglect

## Abstract

Following bi-parietal lesions patient AT showed a severe inability to relocate her attention within a visual field which perimetry proved to be near-normal. An experimental approach with tasks testing visuo-spatial attention demonstrated a shrinkage of A.T.’s attentional visual field. With her visual attention narrowed to a kind of functional tunnel vision, the patient exhibited simultanagnosia (Wolpert, 1924), a symptom previously described in 1909 by Balint under the label of Psychic paralysis of “Gaze”. In striking contrast AT showed an efficient and effortless perception of complex natural scenes, which, according to recent work in normal subjects, necessitate few if any attentional resources.

## 1. Introduction

When studying patients presenting visual deficits the neuropsychologist must take into account three important recent discoveries: the new maps of the visual cortex as recently delimited in vivo by fMRI and PET scanning [34]; the richness of forward and feedback connections; and the existence of three afferent systems: parvo-, konio- and magno-cellular pathways roughly distributed into two cortical streams, a ventral and a dorsal one [24] Both systems analyse the visual input, but for different purposes summarized as recognition and identification for the ventral system, visually guided behavior for the dorsal system. Yet a too strict dissociation between the ventral and dorsal streams is open to debate [24–26].

The dichotomy of the visual system into two streams of processing has been readily accepted by neuropsychologists who have long been familiar with such a dissociation. Indeed they have learned in their textbooks to contrast visual agnosias (alexia, object agnosia, prosopagnosia, achromatopsia, akinetopsia) provoked by temporo-occipital lesions with the Balint's or Holmes's [21] syndromes provoked by bi-parietal lesions. According to a pioneer paper by Balint [1] bilateral damage to the posterior parts of the parietal lobes induces three main impairments: *"Seelenlähmung des "Schauens", optische Ataxie, raümliche Störung der Aufmerksamkeit"*, usually translated as "*Psychic paralysis of "gaze", optic ataxia, and spatial disorder of attention*" (see Harvey in (1). Further studies [3,7,11,18, 21,26–28,35] have shown that the parietal lobes are involved in creating multiple spatial representations and in shifting from one spatial reference point to another.

Even when Balint's triad is present, it often happens that the three symptoms are not equally represented in an individual patient. Here we present patient AT, who sustained a lesion closely similar to the lesion of the classic Balint's patient, i.e. a bi-parietal lesion. However the bilateral lesion in our patient was an occipito-parietal lesion, that is a lesion which also involved the prestriate visual areas above the upper calcarine fissure. If one adopts Balint's terminology, our patient

ISSN 0953-4180/04/$17.00 © 2004 – IOS Press and the authors. All rights reserved has presented both an *optic ataxia and a psychic paralysis of "gaze"*. Leaving aside the description of the massive spatial deficit presented by the patient, the current paper will focus on an experimental approach to the "Schauen" deficit (usually translated as a "gaze" deficit). Like Balint's patient our patient AT exhibited a visual deficit, although perimetric examination showed the visual field to be near normal. A normal visual field does not guarantee that the patient has efficient use of his perceptual abilities (e.g. extinction). Indeed a perimetric examination is based on the detection of small points on a black background, one point at a time. But natural vision is more than a capacity to detect isolated points, it implies the ability to focus and browse in a scene composed of innumerable contrasts of luminance, color, movement and texture.

In this paper we will present several experimental data which validate Balint's pioneer idea that the *Psychic paralysis of "gaze"* is the result of both a weakening and a shrinkage of the attentional visual field.

## 2. Clinical picture

A.T, born in 1953, has been regularly followed since December 1987 when she presented an eclamptic attack during the delivery of a pair of twins. The hypertensive episode provoked a hemorrhagic softening in the territory of both parieto-occipital arteries (branches of the posterior cerebral arteries). Initially, when recovering from coma, AT presented a severe visual deficit similar to cortical blindness. She was so completely disoriented that she could not find her way around her own apartment and could hardly dress herself without help.

For years after the damage, A.T. has presented with a set of diverse symptoms: right-left disorientation, simultagnosia, optic ataxia, constructional apraxia, spatial agraphia, and acalculia. Primary sensory and motor abilities, general attention, language, memory, reasoning, and emotional status could be considered as normal. AT showed no sign of visual agnosia: she could identify objects, animals, fruits and vegetables, images, monuments, faces, and facial expressions. Colour recognition was normal (perfect score on the Farnsworth 15, presented at a 1 meter distance; color naming intact). She easily recognized segmented objects of the Hooper visual organisation test (identifying 19 out of 20 objects drawn from a French customized version of this test).

The body schema was normal and AT could dress herself, albeit slowly and carefully. A year after the stroke she managed to lead a reasonably normal life, taking care of two children and keeping her house without serious problems. Yet AT still faced difficulties with tasks requiring precise and fast control within personal and extrapersonal space, like dressing her children, making up her face, setting a table, inserting prongs of a plug into a power supply socket, cooking, ironing, sewing, catching a ball, piling books, orienting her shoes before putting her feet into them, approaching the first step of a staircase, putting her signature on the right place on a bank cheque, finding a word in a dictionary etc. She walked with her eyes fixating the ground. The slowness in everyday complex actions was striking. Despite many rehabilitation efforts she has remained unable to write fluently (while she spelled normally), to set out figures for calculation, to draw or even copy drawings. Optic ataxia has remained stable, impairing precise pointing and reaching [14,23] especially outside the fovea. AT complained that "her gaze would not reach directly what she wanted to see". Yet eye movements were apparently normal but precise recording of pursuit or saccadic eye movements was not carried out.

AT was no longer able to type spontaneously, although she had previously worked as a secretary. But if one helped her to place her digits on the keyboard, she remained able to type, up to a paragraph of several lines, as long as she could keep her fingers on the keyboard. This seems to have been automatic typing based on motor memory rather than use of the visual display of the keyboard, since she was quite unable to place the letters in their right place on a drawn keyboard.

In the daily behavior there was no apparent sign of right or left spatial neglect. In the line bisection task, accuracy was very poor but there was approximately the same amount of right or left bias occurrences.

## 3. Lesions

The cerebral lesions were quite remarkable on the MR images (Fig. 1). They involved bilaterally a large cortical area between the upper calcarine fissure and the occipito-parietal sulcus. They extended to white matter in the fronto-parietal areas. The cerebellum was normal.The lesions were equally distributed between both hemispheres, almost mirror images of each other. They spared both V1 and V2 (except for a small left upper calcarine polar area), ventral V3, perhaps dorsal

Table 1

| | | | | | | | | | |
|---|---|---|---|---|---|---|---|---|---|
| % coherence | 25 | 30 | 35 | 40 | 50 | 60 | 70 | 80 | 90 |
| % correct | 0 | 0 | 12 | 50 | 79 | 83 | 100 | 87 | 100 |

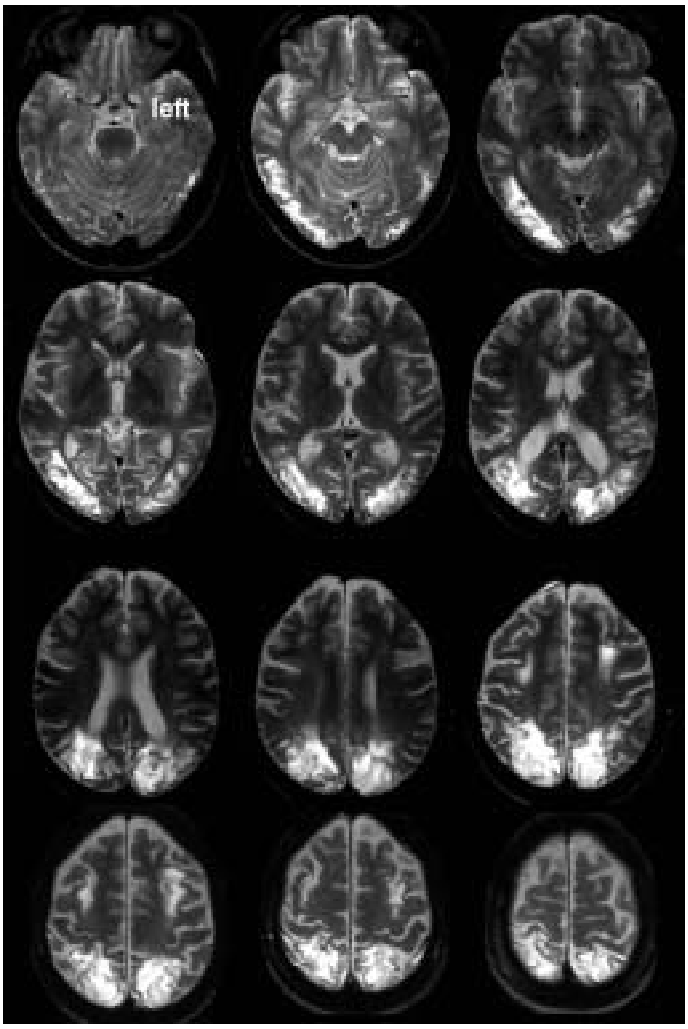

Fig. 1. The MRI horizontal sections show AT's symmetric occipito-parietal lesions (see description in text).

V3, certainly V5 bilaterally). These lesions represent more than the classic watershed softening in the upper parietal areas. They are occipito-parietal. Such a rare type of bilateral lesion is usually encountered following a hypertensive episode during eclampsia. It is attributed to a squeezing of arteries on the tentorium cerebelli provoked by the cerebral oedema.

## 4. Low level vision

Several perimetric examinations over the years of follow-up showed a normal visual field except for a small parafoveal scotoma in the right inferior quadrant (Fig. 2).

Acuity was near normal. Contrast sensitivity tested by low contrast letter charts was found to be in the normal range. Contrast sensitivity functions (CSF), static and dynamic, were near the normal range (Fig. 3).

Stereoscopic vision was fair according to random-dot anaglyph tests.

Color vision was normal according to standard tests (Ishihara; Farnsworth). Also, AT was able to perceive motion based on color contrast.

Orientation perception was quite abnormal: AT completely failed Benton's Judgment of Line Orientation

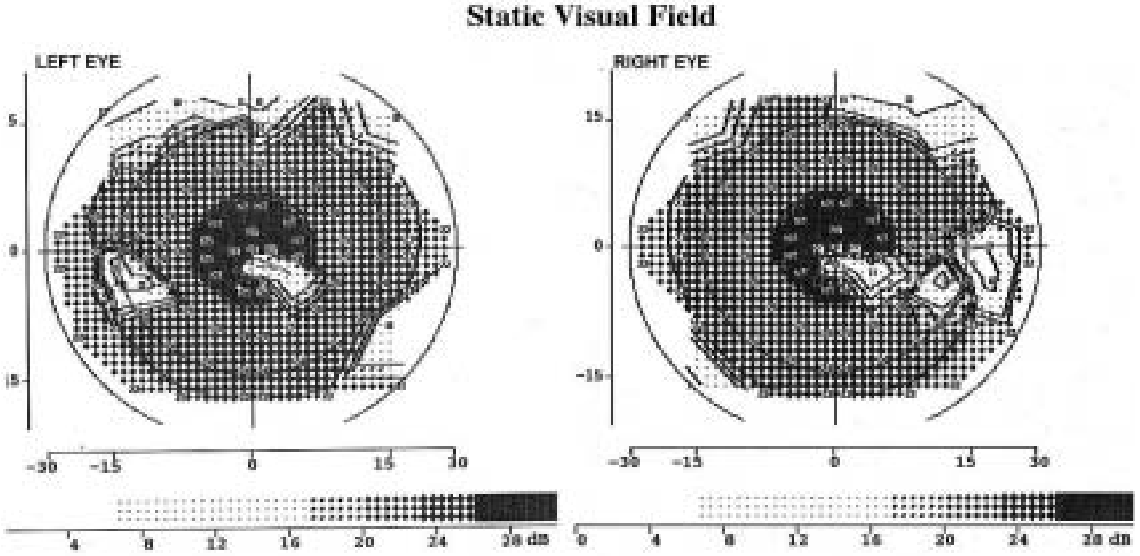

Fig. 2. Perimetric examination showed a small parafoveal scotoma in the right inferior quadrant.

test. But it could be the result of a crowding effect possibly induced by the fan of choice lines. AT was then tested with a staircase procedure. Discs that were ten degrees in diameter filled with gratings of different orientations were presented centered on the fovea. The displays were changed step by step, starting from orientations plus or minus 30° from the vertical or horizontal, until AT accepted the gratings as horizontal or vertical. The mean error for 12 sessions when searching for horizontal orientation was 6.5°± 3.3, while it reached $13° \pm 5°$ for vertical orientation. A control subject (a female secretary of the same age) showed respectively $3.2° \pm 0.8°$ and $2.6° \pm 1.5°$. A similar procedure was used to test her perception of a 45° orientation. The mean difference from this 45° orientation reached $15.3° \pm 5.2°$. This difficulty in judging orientation was incapacitating enough to make AT, for example, unable to insert the prongs of a plug into a power supply socket.

Simple reaction times (ms) to random presentations of a black point in various locations on the horizontal meridian, up to 5° from the center, were: center $487 \pm 31$, right $477 \pm 45$, left $476 \pm 50$. An Anova showed that the RTs were not significantly different in the three locations : $F(2, 85) = 0.337$; $P = 0.71$). These RT values are almost twice as great as 260 ms, which is the accepted normal value for a female of this age. It is worth noticing that the visual reaction time was abnormally high compared to AT's auditory reaction time, which averaged 282 ± 32 ms, that is slightly higher than the accepted normal value of 240 ms. Thus the allocation of attentional resources is quite different in the two modalities.

## 5. High level vision

AT did not complain of visual difficulties in her everyday life. For example, she could easily follow TV programs. Image perception was experimentally tested with complex natural scenes. A series of 35 images (15° wide) of natural scenes (landscapes, monuments, animals. . . ),the same that were utilized in [32], were presented centrally for 50 ms, immediately followed by a mask. AT recognized most images (2 failures, 2 errors, 6 approximations), a performance slightly lower than the ceiling performance of control subjects who nearly recognize all images.

The discrimination of simple dynamic patterns generated by displays of rigidly linked sets of points [15] also requires attention [6]. AT completely failed to recognize the 4 examples of biological movements presented in this manner (a man walking, throwing a ball, playing tennis, lifting a heavy weight).

AT never complained of any problem in perceiving movement. Motion perception was tested with a same-different paradigm: in one visual field dots were moving randomly while in the other visual field dots were moving at varying percentages of coherence. AT was given unlimited time to decide whether the right and left displays were different, i.e. if there was motion on only one side. Her threshold of motion perception was

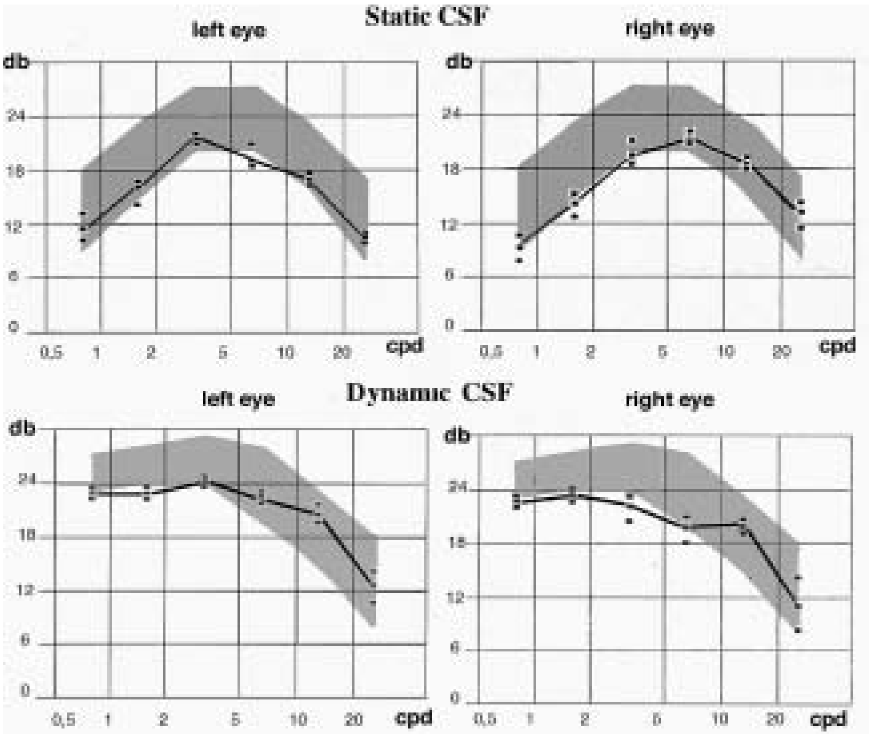

Fig. 3. Contrast sensitivity was low but near normal (gray shading).

found to be at 79% of coherence (Table 1), a performance slightly lower than controls (52% ±8% in our setting).

Recognition of motion-defined shapes (geometrical forms, letters, animal outlines) was easy for AT. Optic flow (up, down, left, right, forward, backward) was also readily perceived, as was the waterfall illusion (motion after-effect).

If low-level motion perception was preserved, higher motion perception tested by apparent motion perception was clearly abnormal: AT was seeing flashing lights when normal subjects would see apparent motion of successively presented light spots (phi phenomenon). This deficit in apparent motion perception was observed in several other patients with unilateral or bilateral parietal lesions [2].

Classic geometric displays leading to illusions (Ebbinghaus, Ponzio, Hering, Muller-Lyer), presented along the years of follow-up (once by session), led to random responses.

## 6. Spatial vision with attention

AT failed to analyse simple static images when spatial attention was necessary (Fig. 4: A,B,C). For example, AT could not locate a point over one of two crossed lines. Nor could she navigate attentionally within a very simple maze to find the correct exit. And she had great difficulty deciding whether a point was inside or outside a simple shape. But in contrast she could identify easily different objects drawn and superimposed in a very restricted area of a sheet of paper (around 1 degree).

Visuo-spatial attention was tested with the classic Posner display of a star appearing randomly in one of three boxes presented on the horizontal meridian [29]. Without any cue (i.e. without previous brightening of one of the boxes) the mean visual reaction time was 472 $\pm$ 59 ms. There was no effect of the star location (right, left or center). In the cued paradigm with 80% valid (target appearing in the brightened box) versus 20% invalid (target appearing in the unbrightened box) the mean RTs were respectively 573 $\pm$ 101 ms and 587 $\pm$ 99 ms. This difference was not significant $F(1, 373) = 1.035$; $P = 0.31$). A similar experiment with a central arrow as a valid or invalid cue led to longer mean RTs: 773 $\pm$ 134 ms for valid cue trials and 752 $\pm 149$ ms for invalid cue trials (paradoxically RT for valid cue trials being longer). Again there was no statistically significant difference between the two types of cueing $F(1, 188) = 0.755$, $P = 0.38$). Ap-

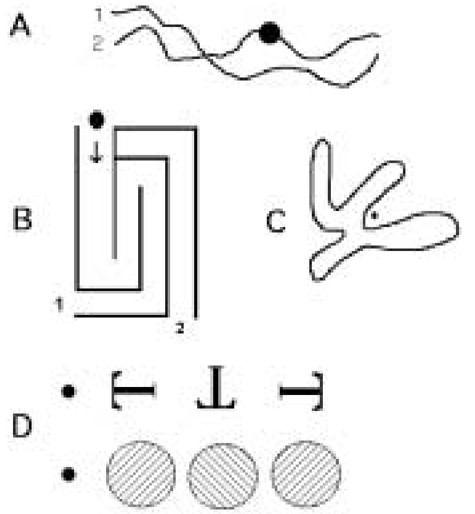

Fig. 4. Four tasks which require spatial attention: A- Deciding if the black point is on line 1 or line 2; B- Finding the exit of a maze; C- Deciding if the black point is inside or outside a complex shape; C- Judging the orientation of the middle letter or middle grid while fixating the black point.

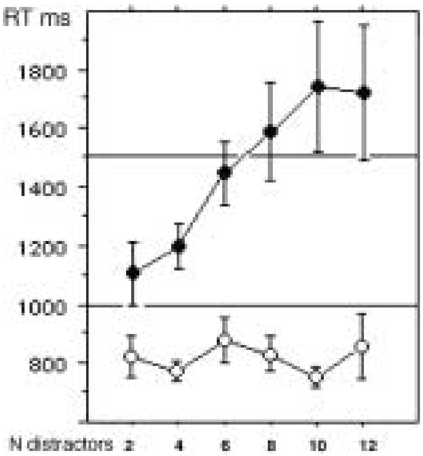

Fig. 5. Decision as to the presence or absence of an odd item within a set of several scrambled discs, on the basis of one parameter (green vs red) or two parameters (small or big, red or green). For one-parameter displays AT's response times remained stable despite the addition of distractors (open circles). For two parameters displays response times were longer and increased linearly with the addition of distractors (solid circles).

parently the mechanisms of covert attention, i.e. shifting attention without eye movements, were no longer available to AT. This fits with the proposal that the parietal lobes are of major importance for covert attention mechanisms [29], though of course this type of chronometric result in only one patient is hardly decisive. In all experiments there was no difference between right and left visual fields.

The classic Treisman tasks [33] were used to contrast automatic perception versus attentive perception, i.e. pop-out vision versus visual search. The task was to detect the presence or the absence of a target within an increasing set of distractors. The targets were identified by their orientation or their color: a horizontal bar among vertical bars, a red bar among green bars. The presentation time was 200 ms. For pop-out displays (two colors or two orientations), AT showed stable performances (response times and percent correct) even with a large number of distractors. On the other hand, performance clearly worsened when a conjunction was involved (two orientations plus two colors): RTs were almost doubled (Fig. 5) and the impairment increased with the number of distractors. Such an RT increase is clearly much higher than that observed in normal subjects.

A Stroop task was designed with three types of displays: the color name printed in the compatible color, on a different color background (compatible); the color name printed in another color over a background of the printed color name (Stroop); the color name printed with a different color over a different color background (different). Displays were centrally presented for 300 ms. AT's performance in naming the color in which the word was printed was compared with her husband's performances (Table 2).

Table 2

| | compatible | stroop | different |
|---|---|---|---|
| AT RT/ms | 8451 | 9948 | 7643 |
| AT % correct | 85% | 45% | 47% |
| GT RT/ms | 1819 | 1767 | 2028 |
| GT % correct | 100% | 100% | 100% |

AT showed an obvious difficulty in managing to perform a task that her husband (GT in the table), who had near the same age and the same socio-cultural level, performed perfectly.

In the Navon task visual attention can play a role in identifying global and local targets in hierarchically structured patterns, focusing on the overall picture or on its letter components. The presentation of large letters composed of small letters allows one to test global vs. local attentional processing. Figures are 'coherent' if the large letter is made up of the same small letters, 'incoherent' in the inverse case. The task was to read the small letter or the large letter. Reading the large letter was very difficult for AT: 15% correct for 100 ms presentations ($N = 32$), 29% correct for 1000 ms presentations ($N = 32$). By contrast, reading the small letter was strikingly easy: 100% correct for a 100 ms

Table 3

| N discs presented | 1 | 2 | 3 | 4 | 5 |
|---|---|---|---|---|---|
| % correct (300 ms) | 50% | 30% | 50% | 50% | 40% |
| % correct (1500 ms) | 100% | 80% | 85% | 60% | 45% |

presentation ($N = 32$). Obviously AT was preferentially seeing the local component rather than the global display.

In an enumeration task [9], the observer must attentionally count a small number of static items in a given display. First, a 10° square mask, made up of a matrix of 10 × 10 juxtaposed 1° discs was presented for 1000 ms. Then, 1 to 5 discs were presented for 300 or 1500 ms at various locations in the surface delimited by the mask. There was no mask after the display of the individual discs. The task was to determine the number of discs presented after the mask.

These results (Table 3) show extremely poor performance, since 5 control observers did show ceiling performances even for the most "difficult" presentation of the displays used (5 discs presented for 300 ms ).

Recognition under crowding conditions was particularly difficult. When AT was fixating a central point she could not easily recognize simple items located 3° out from the fovea when they were flanked by similar items. For example she could not decide if a T was upside down or if an oblique grating was oriented to the right or to the left (Fig. 4D). Again such a task requires covert attention. Reading letters within a context was difficult (Fig. 6). A target letter was presented (300 ms) either isolated or flanked on each side by one other letter. When the target letter was isolated reading was near perfect. When the target letter was between two flankers AT's reading decreased as a function of proximity. By contrast, reading of the right or the left flanker letters was near-perfect.

Tracking several moving objects at the same time cannot be performed by pursuit eye movements. It requires covert attention. AT was presented with 3 to 6 green discs moving randomly in a rectangular area. For each trial, one (or two) discs were identified as targets by briefly turning red. After 2 s, they turned back to green and AT was asked to track the targets either freely or while fixating a central fixation point. After 3 s, all discs stopped moving and AT was required to report which was (or were) the original targets. This setting should have been very easy, since normal controls can track up to 4 target discs embedded among 9 distractors [13]. Performances during repeated sessions remained extremely poor (Table 4). AT was only able to track one slowly moving (1°/s) target disc, and only if she was allowed to follow its movements using foveal pursuit. She completely failed when attentional tracking was required, i.e. while fixating the center or as soon as there were 2 targets).

Table 4

| Targets discs/ totaldiscs | NB trials | Speed | %correct | attention or free tracking |
|---|---|---|---|---|
| 1 disc / 2 | 34 | 1 | 92 | free |
| 1 disc / 2 | 12 | 1 | chance | attention |
| 1 disc / 2 | 24 | 3 | 54 | free |
| 1 disc / 4 | 80 | 2 | 60 | free |
| 1 disc / 5 | 12 | 1 | failure | attention |
| 1 disc / 5 | 12 | 1 | 90 | free |
| 1 disc / 5 | 12 | 2 | 87 | free |
| 1 disc / 5 | 12 | 3 | 53 | free |
| 1 disc / 5 | 12 | 4 | 45 | free |
| 2 discs / 5 | 12 | 1 | failure | free |

According to fMRI studies [8,10,16], attentive tracking triggers parietal activity in normal subjects, suggesting that parietal cortex is involved in continuous "attentional pursuit" of moving objects. Here the complete tracking failure suggests that the posterior parietal cortex not only participates but seems necessary to accomplish this type of attentive tracking.

In the "ball and shadow illusion" [17] the diagonal or horizontal trajectory of the cast shadow of a ball rolling on a checkerboard makes the observer perceive the ball as rolling along a diagonal trajectory or appearing to rise above the checkerboard floor. AT did not perceive this apparent change of trajectory induced by changes of the shadow trajectory, a change readily seen by normal observers. This is not a surprise since the illusion results from the capacity of the observer to watch both the rolling ball and its shadow (with its normal or deviant trajectory). But for AT, the difficulty was precisely one of following two items at the same time as shown by the tracking experiment (see above). This result suggests that the perception of the cast shadow requires some visuo-spatial capacities.

AT could read books, though very slowly. Yet, she often failed to find automatically the first word of the next line and used the left index finger to secure the progression from one line to the next. Reading of words under brief (50 ms) parafoveal presentation conditions was normal in all quadrants except the right inferior one, where perimetry disclosed a scotoma. With a simultaneous presentation (150 ms) of two words, one in each hemi-field, AT read nearly all the words. Yet, when the 150 ms presentation of both words was followed by a mask, AT failed to read most left field words (12% read in left visual field, 87% in the right visual field, $N = 90$ on each side). Thus, this task revealed a left visual extinction, which did not appear in classic

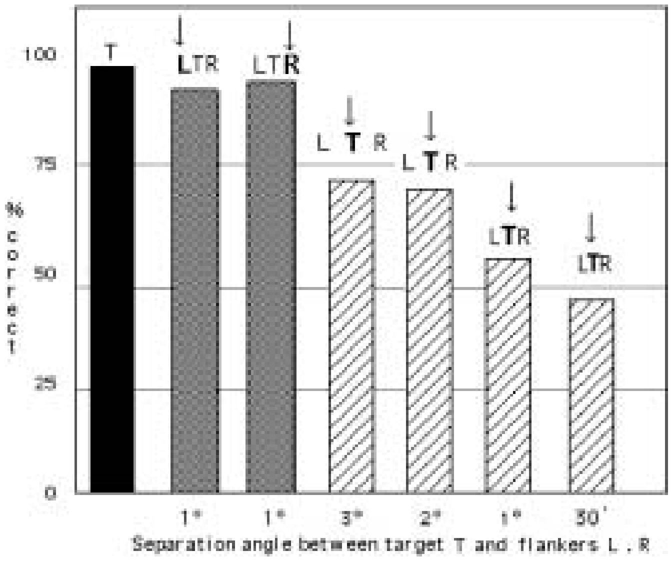

Fig. 6. Reading one letter in three situations: reading the target letter in isolation (T); reading the left (L with arrow) or the right (R with arrow) flanker letter in the three letter display (LTR); reading the target letter (T with arrow) between two flankers (LTR). X axis: distance (in degrees of visual angle) between the target letter and the two distractors; Y axis: percent correct. Ceiling performances for displays T alone, L(TR), (LT)R. For (L)T(R) displays performance decreased regularly with the proximity of the distractors.

neglect tasks. In normal subjects, the performances are exactly the reverse since the right words of the pairs are the first to be ignored when the task is made more difficult with longer words, very short presentations or addition of a mask. AT read easily short sentences (4 words, 20 to 25 letters), presented for 300 ms on the fovea. However, reading of words presented in an unusual orientation could be very difficult for her. Words were presented with orientations varying from horizontal to vertical in steps of 15°. Reaction times increased linearly with orientation ($N = 70$; $R^2 = 0.39$; $p < 0.001$). Also, AT could hardly read any vertical word in a scrabble game context. A more comprehensive study of this will be presented elsewhere.

Counting the number of letters of a long word as well as naming the middle letter of such words was often impossible. Reading of numbers with two or three digits was usually successful but abnormally slow. Reading of four-digit numbers was failed half the time, and, even when successful, was very slow. Reading of numbers of five digits was failed most of the time. In these latter situations, it is necessary to divide the compound items into parts (letters or digits) for a serial visual exploration. Obviously, such segmentation necessitates spatial attentional mechanisms. Thus, there was a striking contrast between reading words through a parallel encoding of the letters and counting numbers through a parsing of individual letters or digits.

## 7. Discussion

In patient AT, the MRI showed that a porencephalic cyst had bilaterally replaced the white matter between the superior lip of the calcarine sulcus and the cingulate sulcus. The cortex around the cyst was reduced to a thin layer. Thus, areas 19 and 7 were nearly destroyed or deprived of afferents and efferents by this unusually large lesion. On the other hand MRI showed a preservation of the whole ventral system, as well as of V5 bilaterally and perhaps of dorsal V3 bilaterally. But beyond V3 and V5 the visual system was so damaged that AT essentially saw the world with the ventral system. In short she presented the exact opposite syndrome of patient SB who was seeing the world without a ventral stream [19].

The first surprise was that AT was able to live a reasonably normal life despite this considerable cerebral damage. She was maintaining a normal alert state and reacted normally to simple stimuli, speech or complex situations. She was able to avoid obstacles when walking or manipulating. Clinically, she had no eye movement impairment. There was no sign that one side of space was preferentially neglected, her deficit in space exploration could rather be described as a bilateral neglect.

AT had a near normal visual field when delimited by classic perimetric assessment. She did not complain of visual problems in her everyday life. At a first glance she seemed to see the world like a normal subject since she had no difficulty seeing movement, colors, objects, faces, landscapes, buildings, and TV programs. She recognized most of 30 experimental images [32], despite a brief presentation followed by a mask. And she could read briefly exposed sentences of up to four words in length. All these capacities are less surprising if one consider that complex natural scenes are processed in the "near absence" of attention [4,20,32].

On the other hand, AT had a very limited field of visual attention, as demonstrated by various tests (crowding tests, counting, attentional tracking, etc.). Thus, AT could not take advantage of her (quasi) full visual field for many visual tasks. As soon as visuo-spatial attention was required, even for tasks as simple as reading a 5-digit number or naming the fourth letter of a 6-letter word, AT showed striking slowness or complete failure. Everyday visual experience requires active vision, in the form of continual attentional scanning, to a much greater extent than, as normal subjects, we may realize [5,6]. Human observers can attend to only a small number of spatial locations at any given time. Subitizing experiments as well as tracking experiments show that one can attend to up to four objects [9,13,30], i.e. there are spatial limits on the allocation of visual attention. Could these limits be lowered by a biparietal lesion? That is what the present case seems to show. In normal subjects, the grain of attention is coarser than the grain of visual resolution [12,13]. According to the present results, AT's attentional resolution was clearly diminished, leading to a shrinking as well as a weakening of the effective visual field (Fig. 7). When AT was engaged in focusing on a small area of a visual display, it was difficult to attract her fovea to another part of the display. Posner et al. [29] raise the possibility of *"three mental operations for a covert shifting of attention: disengagement of attention from its current focus, moving attention to the target, re-engagement of attention to the target"*. Obviously, AT's incapacity to disengage her attention [35] yielded a form of bilateral neglect and "sticky vision". One may sum up AT's deficit by calling it "attentional tunnel vision", or "spotlight vision" or "reduction of ambient vision". For AT, even two items were a crowd. Passive vision was apparently intact while active vision was severely impaired.

These results show that active vision requires processing that can be provided only by the occipito-parietal system, too often reduced to a "where" system.

Theories of perception have proposed a basic distinction between parallel pre-attentive and serial attentive

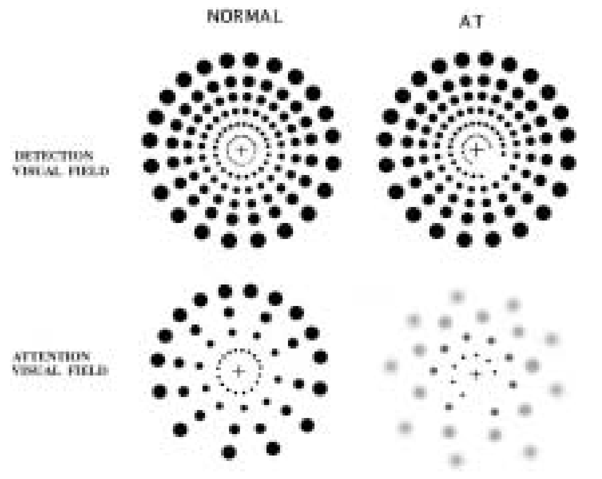

Fig. 7. A schematic representation of the sensory versus attentional visual field in normal subjects and AT (after Intrilligator & Cavanagh [13]). The sensory visual field was almost normal while the attentional visual field seemed severely impoverished.

modes of processing. Patient AT showed a clear deficit of the serial attentive mode of processing, a spectacular example being her difficulty in reading numbers of more than three digits. On the other hand, she has still available the preattentive mode of processing, which permits a normal vision in everyday life and in perception of natural well known images, even briefly presented. Yet her capability of preattentive processing is probably somewhat limited since her performances in the subitizing task and in the letter crowding task (see above) are inferior to normal.

Balint's syndrome was originally defined by three cardinal symptoms: *Optic ataxia; Spatial disorder of Attention; Psychic paralysis of "gaze"*. The symptom optic ataxia has received considerable attention while the two other symptoms of the triad have gained less interest. A careful reading of Balint's original article leads one to think that the *Raümliche Störung der Aufmerksamkeit* (Spatial Disorder of Attention) is what we would now call a left visual neglect. In Balint's own words: *"The patient's attention was always biased towards right space..."*. Regarding the description of the *Seelenlähmung des "Schauens"*, translated in *Psychic paralysis of "gaze"*, it seems rather clear that Balint was describing a deficit for which Wolpert [36] later coined the word simultagnosia. It is important to notice that in the original paper "*Schauen*" ("*Gaze*") is put between quotes (quotes often neglected in references to the paper). "*Schauen*" should not be confounded with moving the eyes, but should rather be extended to include covert attention. Balint's patient had a normal visual field and his eye movements were normal, but he suffered from a concentric shrinking of the attentional visual field, "*The visual field was so narrowed that once a stimulus was in it, those to the right or left were not perceived at all...* "

It is common to describe the perceptual capabilities of the two systems by contrasting the perception for recognition of the temporo-occipital stream with the perception for action of the parietal stream (even if a cross-talk between both systems is always emphasized). The former dichotomy has been illustrated beyond any doubt by many cases, especially DF's case [22], IG's case [26] and SB's case [19]. Yet, AT's visuo-motor impairment is severe but not as severe as would suggest the current dominant view of the visual system [22]. It is striking that, despite her massive bilateral parietal lesion, AT remains able to roughly point and grasp since in the quoted papers [14,23] a statistical analysis was necessary to unveil and illustrate her visuo-motor deficit. Thus, the spared ventral system seems able to replace, at least to some extent, the damaged dorsal system for guidance and control. Indeed, AT was more precise in a pointing task when the response was delayed [23] suggesting some use of information stored by the ventral system. Thus, AT's case confirms the view [28] that the posterior parietal cortex functions as an "automatic pilot" ensuring the on-line fast control of hand guidance. Similarly, the posterior parietal cortex may also function as an "automatic pilot" in the ball tracking task [13] by selecting one "spot" ball as a spatial reference and fixating on its displacement with minimal effort in order to organize a centered tracking of the set of balls that are wandering around.

In the neuropsychological literature, patients with bi-parietal lesions are more often presented for their visuo-motor deficit, the optic ataxia. AT is a good example of this trend since her visuo-motor deficit has already been the main topic of several previous papers [14,23]. Yet AT's deficit cannot be reduced to a visuo-motor deficit. The present work emphasizes a relatively neglected deficit in such patients, that is a visual attentional deficit. This deficit comprises both an inability to see two items at the same time and a striking difficulty to shift the locus of foveation. Thus, when one specifies the dorsal system as being a visuo-motor system, one must not neglect the word "visuo" which points to the perceptual capacities of the parietal lobe. At the risk of a too long label one could designate the dorsal system as a attentional-visuo-motor system.

To summarize AT's neuropsychological problems, one may say that, in addition to an optic ataxia, she essentially presented a Psychic Paralysis of "Gaze" as described by Balint. This label may nowadays appear somewhat obscure and could advantageously be replaced by the more explicit term of simultanagnosia [36]. Our clinical and experimental data illustrate and emphasize that the deficit underlying simultagnosia is a shrinkage and an impoverishment of the attentional visual field (Fig. 7), just as suggested by Balint himself in his pioneer 1909 article.
